# The Delayed Neuroprotective Effect of Methylene Blue in Experimental Rat Brain Trauma

**DOI:** 10.3390/antiox9050377

**Published:** 2020-05-02

**Authors:** Elizaveta E. Genrikhs, Elena V. Stelmashook, Dmitriy N. Voronkov, Svetlana V. Novikova, Olga P. Alexandrova, Mikhail V. Gulyaev, Nickolay K. Isaev

**Affiliations:** 1 Research Center of Neurology, Volokolamskoe shosse 80, 125367 Moscow, Russia; genrikhs@neurology.ru (E.E.G.); voronkovdm@gmail.com (D.N.V.); novikova.s.v@neurology.ru (S.V.N.); aleksandrova.o.p@neurology.ru (O.P.A.); nisaev61@mail.ru (N.K.I.); 2 Faculty of Fundamental Medicine, M.V. Lomonosov Moscow State University, 119991 Moscow, Russia; gulyaev@physics.msu.ru; 3 Biological Faculty, M.V. Lomonosov Moscow State University, 119234 Moscow, Russia

**Keywords:** traumatic brain injury, neuroprotection, methylene blue, microglial cells

## Abstract

After traumatic brain injury (TBI), an increase in dysfunction of the limbs contralateral to injury focus was observed. Using different behavioral tests, we found that a single intravenous injection of methylene blue (MB, 1 mg/kg) 30 min after the injury reduced the impairment of the motor functions of the limbs from 7 to 120 days after TBI. Administration of methylene blue 30 min after the injury and then monthly (six injections in total) was the most effective both in terms of preservation of limb function and duration of therapeutic action. This therapeutic effect was clearly manifested from the seventh day and continued until the end of the experiment—by the 180th day after TBI. MB is known to possess antioxidant properties; it has a protective effect against TBI by promoting autophagy and minimizing lesion volume in the first two weeks after TBI. Studies of the brains on the 180th day after TBI demonstrated that the monthly treatment of animals with MB statistically significantly prevented an increase in the density of microglial cells in the ipsilateral hemisphere and a decrease in the thickness of the corpus callosum in the contralateral hemisphere in comparison with untreated animals. However, on the 180th day after TBI, the magnetic resonance imaging scan of the animal brains did not show a significant reduction in the volume of the lesion in MB-treated animals. These findings are important for understanding the development of the long-term effects of TBI and expand the required therapeutic window for targeted neuroprotective interventions.

## 1. Introduction

Traumatic brain injury (TBI) is a major neurobiological and medico-social problem due to the significant prevalence and severity of medical, social, and economic consequences. The Centers for Disease Control and Prevention estimates that at least 1.7 million TBI cases occur annually in the United States [1], and this rate is continuously on the rise. Functional brain disorders caused by TBI can be very diverse, since they depend on the activation and development of primary and secondary mechanisms of nerve tissue damage [2]. The primary damage occurs as a result of a mechanical damage of the nervous tissue. The secondary damage begins to develop a few minutes after the primary damage and can last for months and probably years as a result of oxidative stress, development of long-term inflammation, disruption of the blood-brain barrier, and progressive damage to the white matter and other metabolic, cellular, and molecular events [3,4]. The secondary damage after TBI is an important problem because it has a long development period. Moreover, the interrelation between early treatment and its effect on the long-term consequences of TBI remains largely unexplored, although this problem is very important for the clinic. It should be noted that many of the already tested neuroprotective drugs aimed at eliminating the consequences of TBI were ineffective. Apparently, to stop the development of multiple cascades of neuron damage during neurodegenerative processes, neuroprotectors should aim at several potential targets for therapeutic interventions at once. One such drug that can be used in a multivalent therapeutic strategy is methylene blue (MB). According to some studies, MB can exhibit antioxidant properties, as well as protects cells from death induced by the action of inhibitors of the complex I of the electron transport chain [5,6,7]. Several studies showed that MB diminishes cerebral damage caused by TBI [7,8,9]. MB is able to penetrate the blood brain barrier following intravenous injection and its concentration in the brain rapidly (approximately in one hour) becomes 20 times greater than in plasma [10]. Methylene blue exerts a neuroprotective effect by promoting autophagy, minimizing lesion volume, behavioral deficits, and neuronal degeneration following mild TBI [8,11]. However, the authors studied the therapeutic effect of MB for only up to 14 days after TBI, whereas, secondary brain damage develops over several months after traumatic brain injury. Studies of the effects of neuroprotection in animal models of TBI over a longer time period are currently scarce. The aim of our work was to investigate the protective effect of MB on the delayed consequences of TBI in a rat model of traumatic brain injury in vivo. We show that for a persistent therapeutic effect of methylene blue, its monthly administration after TBI is required.

## 2. Materials and Methods

### 2.1. Modeling Traumatic Brain Injury

The study was performed on male Wistar rats with body weights of 180–250 g (age 2.5–3 months). In the present work, we used a previously described modification [12] of a model of open focal rat brain trauma [13]. Before the surgery, the animals were anesthetized and fixed on a stereotaxic apparatus [14]. Briefly, to induce TBI, the left part of the skull was trephined above the sensorimotor cortex zone determined according to the atlas [15]. Teflon piston 4 mm in diameter with depth of insertion of 2.5 mm was struck from the height of 10 cm with a 50 g load sliding [16]. Only a skin incision, which was then sutured, was made to sham-operated animals (5 animals). In the first series of experiments, saline solution (5 animals) or MB (1 mg/kg body weight, 5 animals) in saline solution was injected intravenously (vena caudalis) 30 min after the trauma and then monthly (1 injection per month, 6 injections in total). In the second series of experiments, saline solution (5 animals) or MB (1 mg/kg body weight, 5 animals) in saline solution was injected intravenously (vena caudalis) 30 min after the trauma (only 1 injection).

The animals were treated and subjected to experimental procedures in accordance with requirements of the Counsel of the European Community 86/609/EEC on use of animals for experimental studies. All experimental protocols were approved by the Animal Ethics Committee of the Research Center of Neurology (Protocol 2-5/16).

### 2.2. Behavioral Testing

To assess the sensorimotor responses of limbs to tactile and proprioceptive stimulation, we used limb-placing test [17], modified and improved by us [18]. Briefly. The test was comprised of limb-placing tasks: raised above the table, the rat was made to stretch out both of the forelimbs to the table top and move the hindlimbs in the air; the rat with forelimbs placed on the edge of the table resisted, gently pushing toward the edge, as well as turning over and kicking its forelimbs; the animal placed parallel to the edge of the table (the right or the left side or backside) quickly draws alternately taken down limbs paws back to its original position. Behavioral assessments were conducted in a blind manner. Each limb-placing task for each limb was scored as follows: 2 points, test performance is complete; 1.5 points, test performance is complete, but with a slight delay; 1 point, a delayed and/or incomplete performance; 0.5 points, the task is not performed every time, with a large time delay; 0 points, the limb does not perform the task, the lack of response to the stimulation of the limb. Maximum total scores for each side (right or left) before TBI were 12. The test was carried out first before TBI, on the 1st, 3rd, 7th post-injury days, and then monthly up to 180 days.

The grip strength test described earlier [19] was used in a modified form. Briefly, the set for test performance is a fixed crossbar. The grasping reflex and strength of the forelimbs were evaluated. The test clearly demonstrated disorder or recovery of the function. Behavioral assessments were conducted in a blind manner. Each limb by itself was scored as follows: the rat was trying to escape from the hands of the experimenter, kept a tight hold on the crossbar, 2 points; a strong retention of the crossbar by the paw, but let go of crossbar after 6–10 s, 1.5 points; a grip with subsequent retention of the crossbar for no more than 4–5 s, 1 point; grasp, but does not hold the crossbar, 0.5 points; the limb does not grasp, 0 points [20].

The beam-walking test was performed as described earlier [19] using a beam (length of 165 cm tapering from 6 cm to 1.5 cm with a dark compartment to the end) raised above the floor to a height of 90 cm. Animals were trained to properly perform the test for two days prior to testing. At the beginning of the test, a rat was placed in the dark compartment for 1 min. The rat was expected to run the entire length of the tapering track and hide in the dark compartment. To measure the number of misses committed by the limbs contralateral to the damage, a video was recorded and quantitatively assessed in a blind manner.

### 2.3. Morphological Study

The brains of all experimental rats were examined by magnetic resonance imaging (MRI) on the 180th day after the surgery. MRI studies were performed on a 7.05 T BioSpec 70/30 USR MR scanner supplied by Bruker Corporation (Germany) driven by a ParaVision^®^ 5.0 console and equipped with a 105 mT/m gradient amplitude device. T2-weighted images were made in axial projection using “spin-echo” pulse sequence RARE (rapid acquisition with relaxation enhancement) with the following scan parameters: field of view: 2.56 × 2.56 cm; matrix: 200 × 200; bandwidth: 25000 Hz; TR: 4500 ms; TE: 15.51 ms; TEeff: 46.53 ms; RARE factor: 6; number of slices: 20; slice thickness: 0.5 mm; number of averages: 4; total scan time: 15 min. Before the MRI studies, the animals were anesthetized with 1.5% isoflurane mixed with 100% O_2_. The size of the TBI zone was assessed by T2-weighted MR images. For quantitative measurement of the lesion volume, the ImageJ v.1.51j (National Institutes of Health, Bethesda, MD, USA) software was used.

For immunohistochemical and histological studies, on the 180th day after TBI, the animal’s brain was fixed in 4% formalin with phosphate salt buffer for 24 h by the immersion method. To prepare frozen sections, the samples were treated with a 30% sucrose solution and O.C.T. medium (Thermo Fisher, Waltham, MA, USA). A series of frontal sections with a thickness of 10 μm was prepared on a freezing microtome Tissue Tek Cryo 3 (Sakura Finetek, Torrance, CA, USA). Before the immunostaining, tissue sections were processed in a steamer for 15 min in the epitope retrieval EDTA-solution (Dako, pH = 9.0). The slides were incubated overnight with rabbit antibodies against anti-IBA1 (Abcam, Ab178847, 1:150). For visualization, sections were washed with phosphate salt buffer and labeled with secondary goat anti-rabbit CF488 fluorochrome-conjugated antibodies (Sigma, Germany, SAB4600044, 1:150). The preparations were enclosed in a Fluoroshield mounting medium and investigated under the fluorescence Nikon Eclipse Ni-u microscope (Nikon corporation, Tokyo, Japan). Image analysis was carried out on 6–12 sections from each animal, taken with 50–100 μm interval on level of traumatic brain injury. Sensorimotor cortex areas of ipsilateral hemisphere were analyzed using a microscope Leica DMLB (Leica Microsystems, Wetzlar, Germany) with ×10 magnification of the lens. Counting of microglial cells was performed in the marginal zone of damage to the left hemisphere. In accordance with stereological principles, fragments of cells without nuclei and cells adjacent to the borders were not counted.

To evaluate the neurons and myelinated conduction pathways, the sections were stained with luxol fast blue by the Kluver-Barrera method [21] and stained with cresyl violet. The width of the corpus callosum was measured on at least 6 sections on three radial straight lines in the photos taken on a stereo microscope Nikon SMZ18 (Nikon corporation, Tokyo, Japan). For linear measurements in the thickness of the corpus callosum in the photos of the histological sections of the contralateral hemisphere on the 180th day after TBI, the ImageJ software was used. Morphologic assessments were conducted in a blind manner.

### 2.4. Statistics

The one-way ANOVA with Bonferroni post-test was used for statistical analysis of experimental results. Levels of *p* < 0.05 were considered as statistically significant. The results are given as means and standard error of the mean (M ± SEM).

## 3. Results

### 3.1. Effect of Methylene Blue on the Neurological Deficit Caused by Focal Trauma of the Left Sensorimotor Cortex

**Figure 1:**
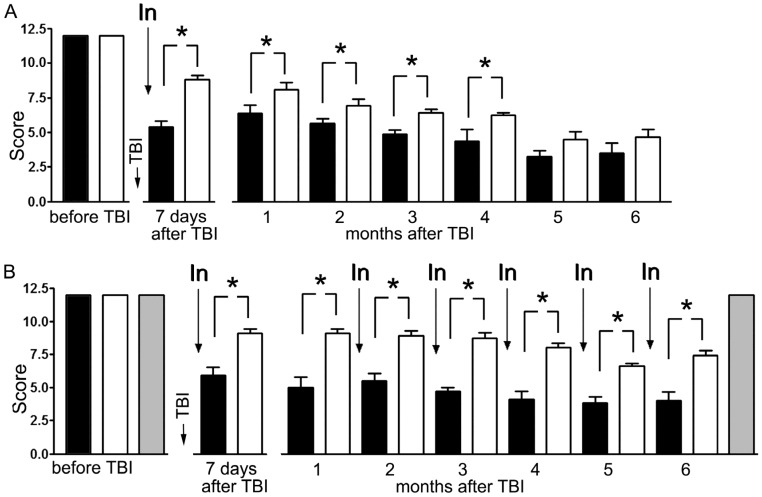
Effect of methylene blue (MB) on the neurological deficit in the right limbs caused by traumatic brain injury (TBI) of the left sensorimotor cortex. The “limb-placing test”. (**A**) A single administration of MB 30 min after the injury; (**B**) administration of methylene blue 30 min after the injury and then monthly (6 injections in total). Animals treated with saline solution after the trauma (black columns), animals treated with methylene blue after the trauma (white columns), sham-operated animals (grey columns). Injection of methylene blue or saline solution (In). * *p* < 0.05.

The limb-placing test showed functional deficits in the right limbs of animals subjected to focal trauma of the left side of the brain, whereas the functions of the left limbs were normal as well as those of all limbs of the sham-operated rats. All animals scored 12 points for the right side before TBI. In the experiment with a single administration of MB, according to the results of the limb-placing test, this parameter on the 7th day after TBI decreased to 5.4 ± 0.4 points in animals that were injected with saline and to 8.8 ± 0.3 points in MB-treated animals (difference in 3.4 points; *p* < 0.001). By 3 months, the difference between treated and untreated animals remained and comprised 1.5 points (*p* < 0.05), whereas after 6 months the treated animals performed the test only slightly better (difference in 1.2 points) (Figure 1A).

In the experiment with the monthly administration of MB, this parameter on the 7th day after TBI decreased to 5.9 ± 0.6 points in animals that were injected with saline and to 9.1 ± 0.3 points in MB-treated animals (difference in 3.2 points; *p* < 0.001). By 3 months, the difference between treated and untreated animals remained and comprised 4 points (*p* < 0.001), and after 6 months the treated animals performed the test significantly better (difference in 3.4 points; *p* < 0.001) (Figure 1B).

**Figure 2:**
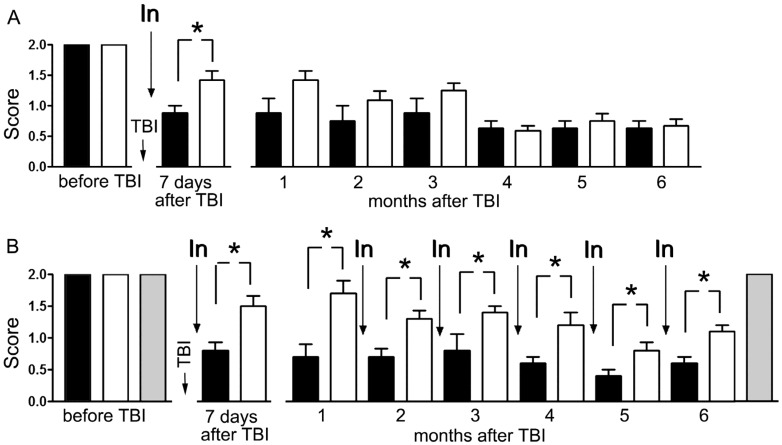
Effect of methylene blue on the neurological deficit in the right forelimb caused by traumatic brain injury (TBI) of the left sensorimotor cortex. The “grip strength test”. (**A**) A single administration of MB 30 min after the injury; (**B**) administration of methylene blue 30 min after the injury and then monthly (6 injections in total). Animals treated with saline solution after the trauma (black columns), animals treated with methylene blue after the trauma (white columns), sham-operated animals (grey columns). Injection of methylene blue or saline solution (In). * *p* < 0.05.

According to the results of another test (grip strength test), all animals before TBI scored 2 points for the right forelimb as well as the left forelimb. The sham-operated rats scored 2 points for all limbs during the entire experimental period. In the experiment with a single administration of MB, this parameter for the right forelimb on the 7th day after TBI decreased to 0.88 ± 0.13 points in animals that were injected with saline and to 1.42 ± 0.15 points in MB-treated animals (difference in 0.54 points; *p* < 0.01). By 3 months, an insignificant difference between treated and untreated animals remained and comprised 0.37 points, while after 6 months the difference was not detected (Figure 2A).

In the experiment with the monthly administration of MB, this indicator on the 7th day after TBI decreased to 0.8 ± 0.12 points in saline-treated animals and up to 1.5 ± 0.15 points in MB-treated animals (difference in 0.7; *p* < 0.001). By 3 months, the difference between treated and untreated animals remained and comprised 0.6 points (*p* < 0.01), and after 6 months the treated animals performed the test significantly better (difference in 0.5 points; *p* < 0.05) (Figure 2B).

**Figure 3:**
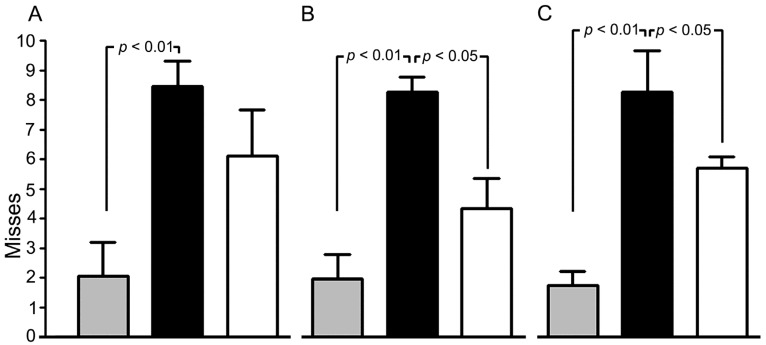
Effect of methylene blue (MB) on the neurological deficit in the right hindlimb caused by traumatic brain injury (TBI). Beam-walking test, the 7th day after TBI (**A**), the 30th day after TBI (**B**), the 180th day after TBI (**C**). Administration of methylene blue 30 min after the injury and then monthly (6 injections in total). Animals treated with saline solution after the trauma (black columns); animals treated with MB after the trauma (white columns), sham-operated animals (grey columns).

The beam-walking test was used to evaluate the function of only the right hindlimb in animals after focal left-sided TBI of the sensorimotor cortex region. The test showed functional deficits in the right hindlimbs of animals subjected to TBI. We assessed the number of misses committed by the contralateral limb on the 7th, 30th, and 180th day after TBI. On the 7th day after TBI, the saline-treated rats had the score of 8.6 ± 0.9 misses, on the 30th—8.4 ± 0.5, on the 180th day—8.4 ± 1.4 misses. Intravenous injections of MB (6 injections in total) diminished the neurological deficit. In MB-treated rats, this parameter was 6.4 ± 1.6, 4.4 ± 1, 5.8 ± 0.4 misses on the 7th, 30th, and 180th days, respectively (Figure 3). Sham-operated rats had the score of 2.1 ± 1, 2 ± 0.8 and 1.8 ± 0.5 misses on the 7th, 30th, and 180th days, respectively (Figure 3).

### 3.2. Effect of Methylene Blue on Microglial Expression and Preservation of Corpus Callosum

**Figure 4:**
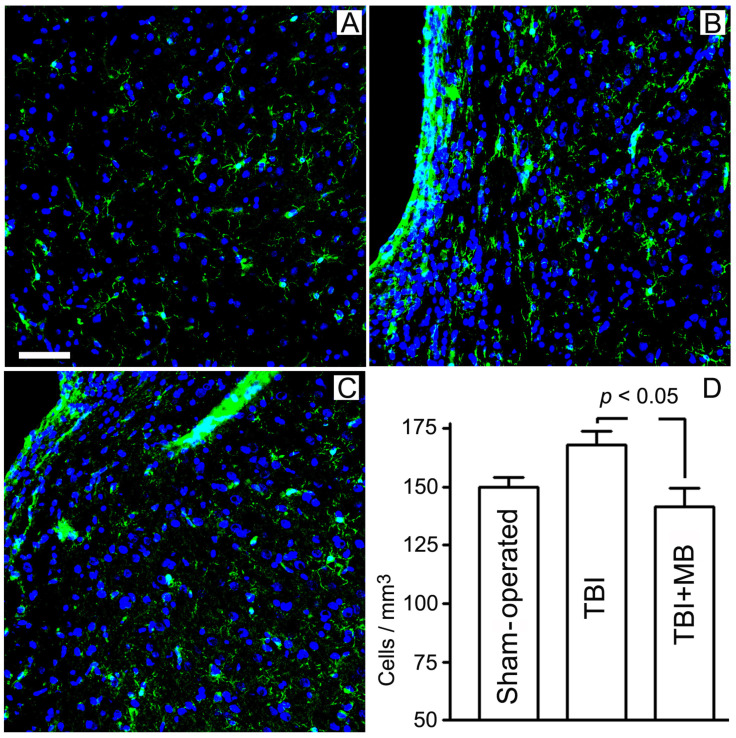
Effect of methylene blue (MB) on the microglial expression in rat cerebral cortex (ipsilateral hemisphere). The 180th day after traumatic brain injury (TBI). Administration of methylene blue 30 min after the injury and then monthly (6 injections in total). (**A**–**C**)—immunohistochemical staining of microglial cells in ipsilateral hemisphere (green fluorescence). The cell nuclei fluoresce blue (DAPI). (**A**)—sham-operated animals; (**B**)—animals treated with saline solution after TBI; (**C**)—TBI + MB. Scale bar, 0.05 mm; (**D**)—quantitative evaluation of microglial density.

On sections obtained from the brain of animals on the 180th day after TBI, we immunohistochemically identified microglia. The results of cell counting in the marginal zone of damage to the left hemisphere showed an increase in the number of microglial cells in untreated animals. While in sham-operated animals this parameter was 150 ± 4 cells/mm^2^, in animals with TBI the number of microglial cells increased to 168 ± 8. Treatment of animals with MB (6 injections in total) significantly reduced the number of microglial cells in the lesion area to 141 ± 8 cells/mm^2^ (Figure 4).

**Figure 5:**
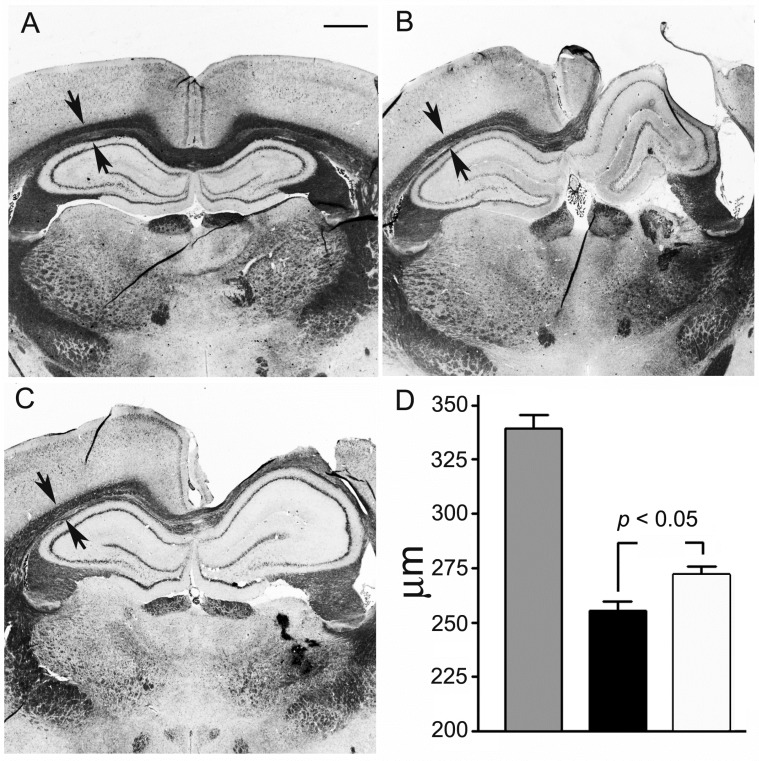
Effect of methylene blue (MB) on the *corpus callosum* thickness in the rat brain hemisphere (contralateral to injury focus). The 180th day after traumatic brain injury (TBI). Administration of methylene blue 30 min after the injury and then monthly (6 injections in total). (**A**–**C**)—Kluver—Barrera and cresyl violet staining on fixed slices. (**A**)—sham-operated; (**B**)—TBI; (**C**)—TBI + MB. *Corpus callosum* is marked by arrows. Scale bar, 1 mm; (**D**)—Quantitative evaluation of the *corpus callosum* thickness, grey columns—sham-operated; black columns—TBI; white columns—TBI + MB.

According to the obtained histological data, the delayed effects of TBI were also manifested in the destruction of the corpus callosum in the left hemisphere and a decrease in the thickness of the corpus callosum in the contralateral hemisphere on the 180th day after TBI. If its thickness was 339 ± 6 µm in the right hemisphere of sham-operated animals, the thickness of the corpus callosum in animals after TBI decreased to 255 ± 4 µm in the contralateral hemisphere. The treatment with MB (6 injections in total) contributed to a greater preservation of the corpus callosum in the contralateral hemisphere, where its thickness was 272 ± 3 µm (Figure 5).

### 3.3. Brain Magnetic Resonance Imaging

**Figure 6:**
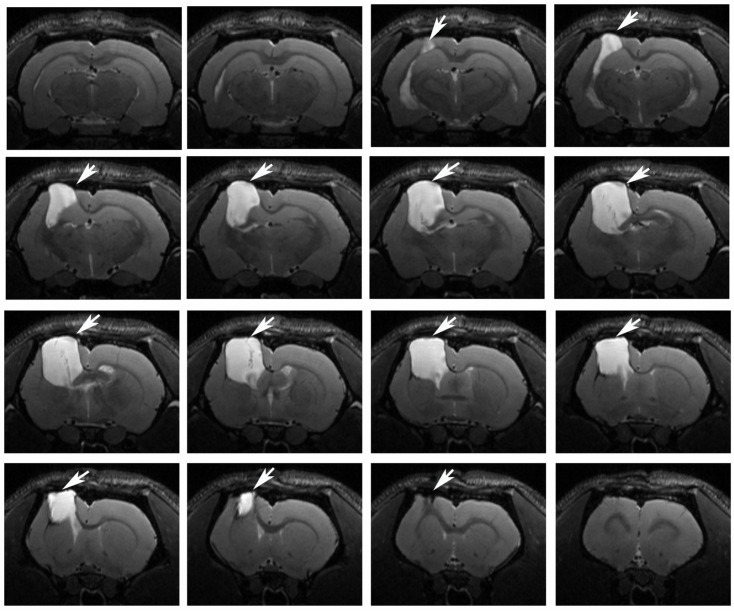
Magnetic resonance images of one experimental rat brain on the 180th day after traumatic brain injury. The left hemisphere, corpus callosum, cortex, and striatum are damaged (the damaged zone is indicated by an arrow).

The long-term effects of traumatic brain injury on the size of the lesion resulting from unilateral focal TBI in rats were investigated using the MRI method. On the 180th day after surgery, T2-weighted MR images detected a clear focus of injury in the sensorimotor cortex, in a large part of the corpus callosum, a damage to the striatum and a dilatation of the lateral ventricle in the left (injured) hemisphere of the brain (Figure 6). Based on MR images of the brain, when the rats were injected monthly, the morphometry of the damaged area did not show a significant difference in the volume of the focus in groups of animals that were injected with MB or only saline. The average size of the focus in untreated animals was 41 ± 3 mm^3^, in treated ones—44 ± 3 mm^3^.

## 4. Discussion

Immediately after the primary brain damage caused by TBI, the secondary damage begins to develop. It can last a very long time and is an important problem, since long-term consequences may not only cause to a deterioration in the neurological status of patients, but also lead to an increased risk of developing neuropathologies, such as Alzheimer’s disease, Parkinson’s Disease, amyotrophic lateral sclerosis [22,23,24,25].

To investigate the long-term consequences of traumatic brain injury and the protective effect of MB on them, we used a model of one-sided focal TBI in the sensorimotor cortex of the left hemisphere of rats [26]. The advantage of this model is that the damage can very accurately be inflicted onto certain areas of the brain using a stereotaxic system. This model makes it possible to study the development of the damage at the histological and functional levels as well as the process of inflammation [27].

The brains of the animals were examined in the long-term period after the injury (six months later). According to the obtained MRI data, by that time a distinct lesion was formed in the left (damaged) hemisphere of the brain, in which the sensorimotor cortex and most of the corpus callosum were destroyed; the striatum was also damaged. It should be noted that an increase in microglial density was observed in the marginal zone of the destruction focus compared to the control animals, indicating the presence of a long-term inflammatory process in the damaged hemisphere in animals with TBI, since the activation of these cells mediates the release of pro-inflammatory mediators (cytokines, chemokines, interleukins, tissue necrosis factor, etc.) [28].

Unfortunately, the literature data on the long-term neuroprotective effects of MB (three or more months) in modeling TBI are rather scarce at the moment. It was previously shown that minocycline administered three times (five minutes, three hours, and nine hours after TBI) reduces the histopathological effects of trauma in mice three months after TBI [29]. Administration of dipeptide mimetic of Nerve Growth Factor after TBI (from first to fourth and from seventh to tenth days after TBI) reduced the impairment of the motor functions of the limbs. This therapeutic effect was markedly pronounced until the end of the experiment—by the sixth month after TBI [30]. A relatively brief exposure to environmental enrichment after experimental traumatic brain injury confers long-term cognitive benefits that last for at least six months after rehabilitation [31]. In our experiments, the monthly intravenous administration of MB after the injury reduced the impairment of the motor functions of the limbs from 7 to 180 days after TBI. A statistically significant improvement in limb function in animals was shown using three different tests: limb-placing test, beam-walking test, and grip strength test. If the protector was administered once, the therapeutic effect was gradually decreasing, becoming insignificant after 120 days of the experiment. A significant deterioration of the limb function was observed in untreated animals compared with the treated animals, which can be mediated not only by primary destruction, but also by secondary subcortical atrophy accompanied by a damage to myelinated axons [32]. This is supported by the fact that degeneration of the corpus callosum was observed both in the damaged hemisphere of the studied animals and on the contralateral side, where the thickness of this structure was significantly reduced. The axons that comprise the corpus callosum are located predominantly in the transverse direction, connecting the symmetrical parts of the opposite hemispheres. However, some axons also connect asymmetrical parts of the opposite hemispheres, for example, the frontal gyrus with the parietal or occipital gyrus, or different parts of the same hemisphere. Local damage to one hemisphere can lead to the development of diffuse damage in various regions of the brain. We showed that treatment with MB statistically and significantly reduced the extent of damage to the corpus callosum. In addition, we showed that the monthly administration of MB significantly prevented an increase in the density of microglial cells in the ipsilateral hemisphere for up to 180 days after TBI. It is well known that glial cells, including microglia, are activated and involved in tissue damage in the peri-lesion regions after traumatic brain injury [33]. Despite successful neuroprotection when MB was repeatedly administered to animals, which was manifested in a decrease in the neurological deficit in injured animals, a decrease in microglia expression in the marginal lesion zone and a reduction in degenerative processes in corpus callosum were observed. Microglia are macrophage-like cells in the brain that are involved in the brain’s innate immune responses and inflammatory processes. These cells change their morphology and phenotype, and become activated when brain damage occurs. Long-term activated microglia and other cells involved in the inflammatory process produce nitric oxide which can cause neuronal death [34,35]. Nitric oxide exerts a toxic effect by inhibiting the complex IV of the mitochondrial respiratory chain, which leads to a damage of the aerobic energy metabolism vital for neurons. MB is capable of reducing NO toxicity by enhancing expression of the complex IV [36].

An important issue is the degree of toxicity of a therapeutic drug. In our study, the MB dose administered to rats was one milligram/kilogram. For these animals, a dose of 20 mg/kg was shown to be harmless, while all physiological changes were reversible within 30 min after administration [37]. Currently, MB doses used for the treatment of methemoglobinemia in humans are about one to two milligrams/kilograms [38,39]. However, this substance at a concentration of two milligrams/liters was shown to exert toxicity on annulus fibrosus cells in vitro [40]. Thus, further research is required to determine possible toxic concentrations of MB.

The study of rat brains using MRI did not find a significant difference in the volumes of foci in MB-treated and untreated animals. However, earlier in the study of the neuroprotective action of MB 7–14 days after TBI, MB was shown to reduce the volume of the focus [11,41]. At the same time, a previously conducted study of delayed (one to six months) TBI-caused damage of brain structures did not find a correlation with neurological deficits [42]. The development of the focus in TBI is known to take a very long time [43], whereas inflammatory and degenerative processes are inextricably associated with oxidative damage [44,45]. Thus, apparently, the neuroprotective effect of MB during prolonged development of the secondary damage is primarily aimed at rehabilitation of diffuse damage as well as reduction in inflammation and therefore in oxidative damage to tissue.

## 5. Conclusions

Thus, our data demonstrate the neuroprotective effect of methylene blue; however, a single injection of this drug is insufficient for the development of long-term neuroprotection, its monthly administration to animals with TBI is required. These findings have importance for the understanding of the development of long-term consequences of TBI, and expand the required therapeutic window for targeted neuroprotective interventions.
